# Effects of Cognitive Load on Trusting Behavior – An Experiment Using the Trust Game

**DOI:** 10.1371/journal.pone.0127680

**Published:** 2015-05-26

**Authors:** Katarzyna Samson, Patrycjusz Kostyszyn

**Affiliations:** 1 The Robert B. Zajonc Institute for Social Studies, University of Warsaw, Warsaw, Poland; 2 University of Social Sciences and Humanities, Warsaw, Poland; Ecole Normale Supérieure, FRANCE

## Abstract

Last decades have witnessed a progressing decline of social trust, which has been predominantly linked to worsening economic conditions and increasing social inequality. In the present research we propose a different type of explanation for the observed decline – cognitive load related to technological development and the accelerating pace of modern life. In an experimental study participants played the trust game while performing one of two different secondary tasks – listening to a disturbing noise or memorizing a sequence of characters – or with no additional task in the control condition. Results show that in both cognitive load conditions participants expressed significantly less trust in the trust game than in case of no cognitive load. Additionally, when cognitive resources were limited, participants’ behavior was more impulsive than when their resources were fully available.

## Introduction

Trust matters. For an individual, trust is a prerequisite for the emergence of a healthy personality and satisfying interpersonal relationships [1–5]. For a group, it promotes cooperation, improves coordination, sustains social order and permits beneficial long-term exchanges [6–10]. As a building block of social capital, trust is important for the stability of democracy and general wellbeing of its members [11–16]. It is also indispensable in finance and necessary for the efficient functioning of modern economies [17–20]. The more complex and dynamic social and economical relations are becoming, the more trust it needed as a lubricant to keep them running [21].

It is thus especially alarming that all around the world trust is declining. Trust in others, as well as confidence in societal institutions, are at their lowest point in over three decades, according to data from two nationally representative surveys in the United States—the General Social Survey of adults and the Monitoring the Future survey of 12^th^ graders—[22]. Similarly in Europe, in the last decade trust has been successively falling [23]. In all advanced industrialized democracies trust towards the government and political institutions has been falling since the late 1960s [24]. What are the reasons for such crisis of trust?

The decline of trust has been linked to a number of phenomena, but mostly to worsening economic conditions [22, 25–27]. Economic inequality is the strongest determinant of a lack of trust, as it hinders the creation of bonds in a society—the greater the inequality gap, the less likely that both the advantaged as well as the disadvantaged will consider themselves part of a moral community [27]. Trust is also negatively related to the size of the national debt and unemployment levels [28]. Other factors that have been shown to contribute to erosion of trust are structural changes in the society [15,16, 29,30]. Increased social and geographic mobility, more ethnic and racial diversity, presence of women on the labor market, changes in the traditional model of family and individuation of leisure weaken bonds holding individuals and communities together and, in turn, erode both interpersonal and political trust. Similarly, the decline of trust has been linked to the rapid rise of materialistic value orientation that undermines views about the trustworthiness of others [31]. At an individual level, strongest factors reducing trust are a recent history of trauma, belonging to a group that historically has been discriminated against, lack of economic success, and living in community which is racially diverse or characterized by a high degree of income disparity [25].

### Cognitive overload

Our society is developing at a great speed and the social and technological changes of the last decades are not without consequences for people’s cognitive functioning [32–34]. The consumption of media is continuously growing. Averaged across all media sources, media delivered in bytes is increasing at a rate of 18% per year [35]. It is estimated that in 2015 Americans will consume on average fifteen and a half hours of both traditional (TV, radio, voice telephony) as well as digital media (tablet computers, mobile gaming devices, smartphones, mobile video) per person per day—and this calculation does not include media consumed in the workplace. While absolute time spent with media is increasing, time spent multitasking with media is growing even more rapidly. Within the first decade of our century, there has been a 120% increase in the time that youth media multitask [36]. British teenagers and young adults on average squeeze 14 hours of media activity into 9 hours of real time [37]. More complexity in our environments along with the omnipresence of information and increased multitasking lead to a state of perpetual cognitive overload [38,39].

Independently of its source, cognitive load decreases performance in all types of tasks [40–42]. It also affects decision-making strategies. Under cognitive load people are more impulsive and less analytic [41, 43], they tend to omit available information [44,45], are more likely to use decision heuristics [46], and fall prey to cognitive biases [47,48]. Cognitive load also decreases self-control [49,50] and the willingness to take risks [51]. This happens because under conditions of cognitive load less resources are available to reflect on a decision, what makes analytical processing more difficult and leads to using cognitive shortcuts.

In the present research we hypothesized that cognitive load may be a factor contributing to the decline of trust observed worldwide in the last decades. Trust is a process that has both social as well as cognitive components—it is not only a belief about the intentions of others but also a rational calculation of probability concerning a specific person in a specific situation [3, 52–58]. We therefore expected that under conditions of cognitive load people would express less trust than in case of a full availability of cognitive resources. We also expected that under conditions of cognitive load the trusting process would be more impulsive, i.e. to a greater extent dependent on immediate, situational cues, as compared to conditions with a full availability of cognitive resources. The results confirmed our hypotheses.

## Materials and Methods

### Ethics Statement

All procedures were approved by a Research Ethics Board at the Robert B. Zajonc Institute for Social Studies, University of Warsaw. Potential participants in the study were informed about its general aim, duration, outline of the procedure and remuneration. They were also told that participation is voluntary, that they can withdraw from the study at any point without telling the reason, and that all data is anonymous and will only be used for reasons related to this study. All participants signed an informed consent form prior to participation.

### Participants

The study was conducted among 90 University of Warsaw students (73% female) aged 19–25 (*M* = 21.63, *SD* = 1.50). They received a small remuneration for participating in the study (ca. 1–2 euros in local currency, exact amount depending on their decisions in the study).

### Measures

To measure trust we used *the trust game* [59]—an economic game, in which two players can increase their wealth through the expression and reciprocity of trust. At the outset of the game both players are endowed with some amount of money. The first player decides how much of their initial endowment they would like to send to the second player, knowing that the whole transfer will be tripled when the other person receives it. Then, the second player returns any fraction of currently possessed money (i.e. the initial endowment enlarged by the received transfer) to the first player. After both players have made their decisions, a round of the trust game ends. The amount of money sent by the first player is the measure of their trust toward the partner, while the amount returned is a measure of the second player’s trustworthiness. The trust game is currently the most common behavioral measure of trust [60,61].

In this study the players’ initial endowments in each round were 10 units of experimental currency. The game consisted of 10 rounds, but the participants didn’t know how many rounds there would be. After each round the players’ money was transferred to their accounts in the game and the next round started with new initial endowments of 10 units of experimental currency per person. After the game ended experimental money from the accounts was exchanged into real currency at a rate of 25:1 and given to the participants as remuneration for their participation in the study. Participants could monitor both players’ account balances at all times.

The trust game was administered online using dedicated software TGAME, which allows controlling the behavior of one the players by replacing it with a computer strategy. All participants played the role of first players, while the computer always followed the same predefined scenario of the second player’s behavior: in the first “trust-building” stage of the game (rounds one through three) it returned 50% of the money it had; in the second “trust-violation” stage (round four) it kept all the money and returned nothing to the trustor; in the third “trust-recovery” stage (rounds five through ten) it again returned 50% of possessed money. To decrease the probability of uncovering that the second player is in fact imitated by a computer, in all rounds except the fourth (violation of trust) there was a random variability in a 10% range added to its decisions.

### Cognitive load manipulation

To impose cognitive load we used two distinct manipulations, the effectiveness of both of which was confirmed in previous research. In case of the first cognitive load manipulation—memory load condition—participants were asked to memorize a password-like string of seven characters including letters, numbers and symbols [62–65]. The second manipulation involved exposing participants to a disturbing noise (noise load condition). Noise has a moderate but deleterious effect on performance capacity and cognitive tasks, such as the trust game administered in this study, are known to be among the most vulnerable to noise effects [66]. The noise used in the present study was a mix of two musical tracks recorded simultaneously of which one was playing backwards.

### Procedure

When participants arrived at the lab, they were randomly assigned to one of three experimental conditions: memory load, noise load, or no cognitive load condition. Before the primary task begun we administered the cognitive load manipulations. In the memory load condition participants were presented a string of characters and asked to memorize it until they would be asked to write it down. In the noise cognitive load condition they received headphones that emitted a noise and were asked to keep them on until instructed to take them off. In the no cognitive load condition participants did not receive any secondary tasks. Next, all participants played the trust game. When the trust game was over, participants in memory load condition were instructed to write down what they remembered of the password and in noise load condition they were told to take off the headphones.

After the trust game and the cognitive load manipulation we also measured participants’ propensity to trust and distrust (a set of generalized beliefs about human nature that determine an initial attitude when interacting with strangers) and their cognitive capacities. Propensity to trust and distrust was measured using Eisenberger’s Expectational Trust Scale (it consists of two subscales—propensity to trust and propensity to distrust) translated by Rozycka and Wojciszke [67]. Cognitive capacities were measured using an abridged version of the advanced Raven’s Progressive Matrices Test [68]; the abridged version included every third matrix from the second series: nos. 1, 4, 7, 10, 13, 16, 19, 22, 25, 28, 31, 34; it had a 10-minute time limit.

When the study was over participants were thanked, carefully debriefed and given their remuneration. The study was conducted during the daytime (10am-5pm) to avoid large differences in participants’ levels of fatigue. The procedure took about 20–30 minutes.

## Results

### Descriptive statistics

We first compared the descriptive statistics of all measured variables (except the dependent variable trust) in three experimental conditions. There were no differences between experimental conditions in terms of age, gender distribution, propensity to trust, propensity to distrust or cognitive capacities. Means, standard deviations and results of group comparison analyses are presented in Table 1. Trust, as measured using the trust game, did not correlate with either propensity to trust, *r*(89) = .17, *p* = .11, propensity to distrust, *r*(89) = -.13, *p* = .21 or cognitive capacities, *r*(89) = .04, *p* = .71.

**Table 1:** Descriptive statistics of measured variables in each experimental condition along with appropriate tests (ANOVA or chi-square, depending on measurement level) to verify differences between the three conditions in means/frequencies.

Variable	No cognitive load	Memory load	Noise load	Group differences comparison
Gender	77% F	70% F	73% F	χ^2^ (2) = 0.34, *p* = .84
Age	*M* = 21.37; *SD* = 1.45	*M* = 21.83; *SD* = 1.26	*M* = 21.70; *SD* = 1.76	*F*(2,87) = 0.76, *p* = .47
Propensity to trust	*M* = 20.90; *SD* = 4.79	*M* = 20.13; *SD* = 5.61	*M* = 18.10; *SD* = 6.02	*F*(2,87) = 2.01, *p* = .13
Propensity to distrust	*M* = 41.27; *SD* = 11.42	*M* = 46.07; *SD* = 13.32	*M* = 43.17; *SD* = 12.03	*F*(2,87) = 1.16, *p* = .32
Cognitive capacities	*M* = 7.13; *SD* = 2.91	*M* = 6.13; *SD* = 3.01	*M* = 6.57; *SD* = 2.96	*F*(2,87) = 0.86, *p* = .43

### Manipulation checks

In the memory load condition we verified that participants really made the effort to remember the password—73% recalled the password flawlessly, another 20% made only one mistake and only 7% made two or more mistakes. In the noise load condition we checked that the track in the headphones was playing throughout the duration of the manipulation and that participants didn’t reduce the volume.

### Differences in trust

**Fig 1:**
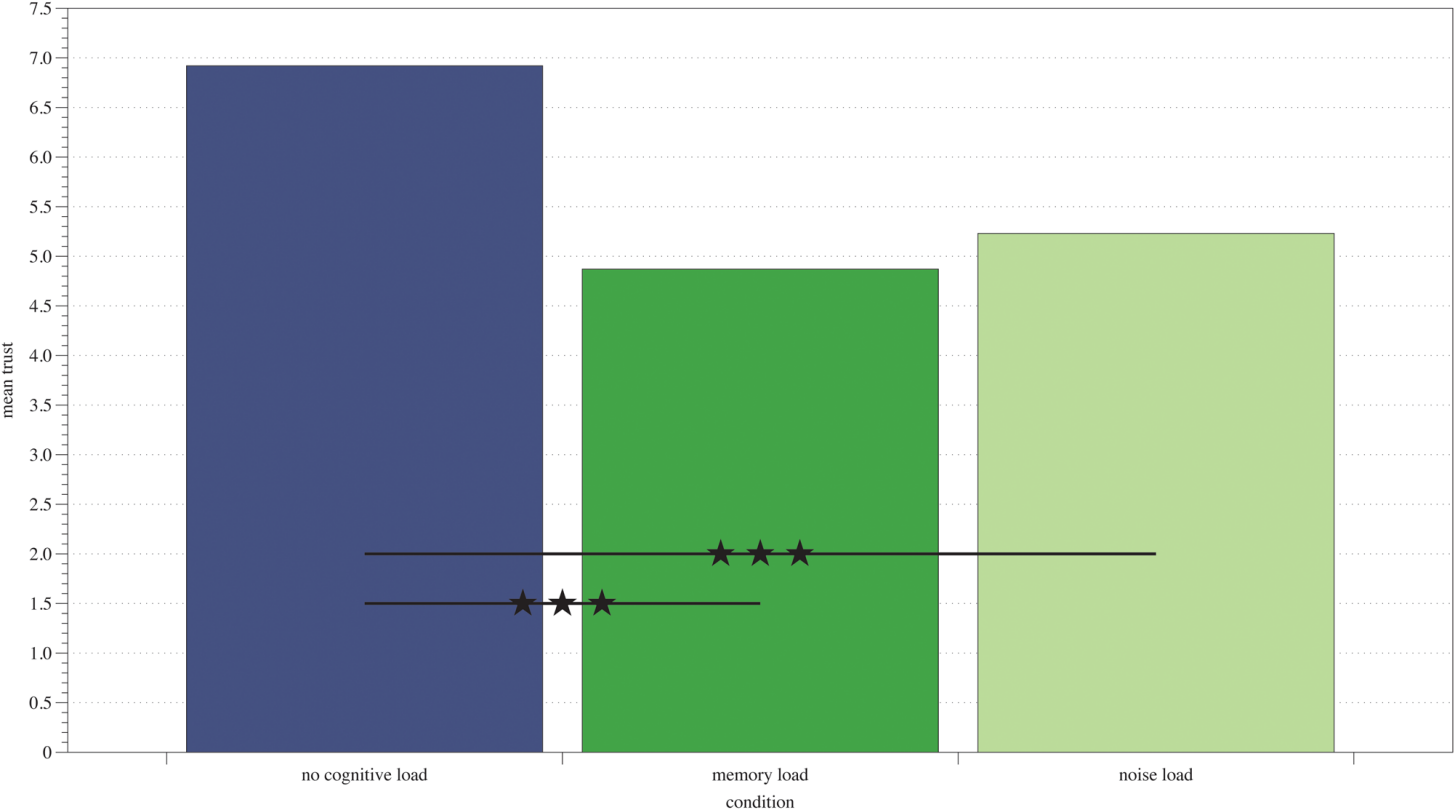
Overall means of trust in the trust game in three experimental conditions. *** *p* < .001.

To verify if levels of trust expressed in the trust game varied between experimental conditions we conducted a repeated measures ANOVA with cognitive load as a between-subject factor and trust in each of the rounds of the trust game as dependent variable. Mauchly’s test indicated that the assumption of sphericity had been violated, χ(9) = 198.29, *p* < .001, therefore degrees of freedom were corrected using Greenhouse-Geisser estimates of sphericity (ω = .60). Results showed that cognitive load indeed affected the levels of trust expressed toward the partner, *F*(2,87) = 10.25, *p* <.001, ηp^2^ = .19. Analysis of Helmert contrasts showed that in in the control condition with no cognitive load trust was higher, *M* = 6.92, *SD* = 1.95, than in case of both cognitive load conditions, *p* < .001. The difference in trust between the noise load condition, *M* = 5.23, *SD* = 1.70, and the memory load condition, *M* = 4.87, *SD* = 1.95, was not significant, *p* = .46. Means of trust in each experimental condition are presented in Fig 1.

**Fig 2:**
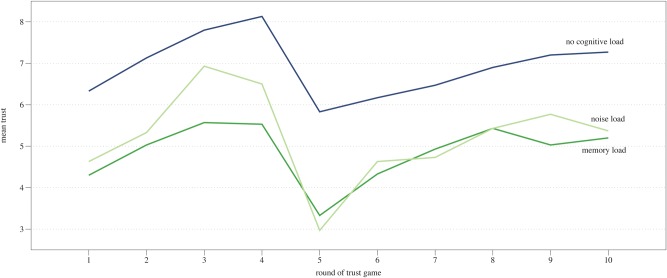
Means of trust in each round of the trust game in three experimental conditions.

Within-subject analysis showed that the average level of trust changed between the rounds of the trust game—across all experimental conditions trust started off at a medium level, increased in the next three rounds, then dropped rapidly in the fifth round and starting from the sixth round increased again to stabilize in the ninth round at a slightly greater value than in begun with, *F*(5.43, 473.10) = 12.98, *p* < .001, ηp^2^ = .13. Repeated contrasts indicated significant differences in mean levels of trust between the first and the second round, *F*(1,87) = 8.53, *p* = .004, ηp^2^ = .09, the second and the third round, *F*(1,87) = 17.99, *p* < .001, ηp^2^ = .17, the fourth and the fifth round, *F*(1,87) = 58.21, *p* = .004, ηp^2^ = .40, the fifth and the sixth round, *F*(1,87) = 12.60, *p* = .001, ηp^2^ = .13, and the seventh and the eighth round, *F*(1,87) = 4.52, *p* = .04, ηp^2^ = .05. These differences follow the assumed pattern stemming from the preprogrammed behavior of the trustee—trust building, trust violation and trust recovery stages. Changes in average levels of trust did not differ between experimental conditions (Fig 2), as the interaction between cognitive load and the round of the trust game was insignificant, *F*(10.88, 473.10) = .72, *p* = .72.

When we adjusted the analyses for propensity to trust, propensity to distrust and cognitive capacities, the pattern of results did not change. Detailed results are available upon request.

### Impulsiveness of decisions

To verify if cognitive load affects the impulsiveness of trust game behavior we first operationalized the concept of impulsive and strategic decisions in the trust game. We operationalized an impulsive decision as one that is guided by the most immediate cue concerning the trustworthiness of the partner, i.e., partner’s last move. This way if the partner was trustworthy in all preceding rounds but betrayed our trust in the last one, an impulsive decision would be not to look on the overall positive history of the interaction but to punish them with distrust for their betrayal. For each trust game decision in rounds two through ten we found the partner’s decision that immediately preceded it and called this predictor *impulse*. Then we operationalized a strategic decision as one that follows an initially adopted strategy, independently of the actual history of the interaction. Examples of such strategies could be “my best chance at getting the most out of the situation is to always trust” or “never trust a stranger”. We assumed that applying a strategic approach should result in an auto-correlated time series of a participant’s own decisions. For each trust game decision in rounds two through ten we computed the average value of that person’s trust in all preceding rounds of the trust game and called this predictor *strategy*.

We then conducted multiple regression analyses using the enter method of including variables in the model, with trust in a given round of the game as dependent variable (rounds two through ten) and two standardized predictors: impulse and strategy, independently for both cognitive load conditions and the no cognitive load control group. Results showed that when cognitive resources were limited, either through a memory task or a disturbing noise, impulse was a stronger predictor of trust than strategy. In the control condition with no cognitive load it was strategy that was more strongly related to trust than impulse. Regression coefficients are presented in Table 2.

**Table 2:** Effects of impulse and strategy on trust in no cognitive load and cognitive load conditions.

	No cognitive load		Cognitive load	
Variables	B	SE	B	SE
impulse	0.81***	.16	1.36***	.11
strategy	1.19***	.16	0.84***	.11
F	70.88***		189.59***	
R^2^	.35		.41	

*** p < .001

## Discussion and Conclusions

In the present research we investigated the effect of cognitive load on trust expressed in a dyadic interaction. Participants played the trust game with a preprogrammed computer strategy that imitated the behavior of a second player. At the same time they either performed a secondary task that engaged some of their cognitive resources—keeping a string of characters in their memory or listening to a disturbing noise on headphones—or not. As predicted, cognitive load introduced by secondary tasks decreased the amount of trust participants placed in their interaction partner, independently of the type of cognitive load manipulation. Moreover, when cognitive resources were limited, participants’ behavior was more impulsive than when their resources were fully available, i.e., partner’s last move was a stronger predictor of behavior than own strategy until that moment.

This work has important theoretical implications regarding the declining indicators of social trust around the world [22, 24]. Our results indicate that living under conditions of almost permanent cognitive load may be a factor contributing to the progressing decrease of social trust. As the pace of modern life is accelerating, people’s cognitive resources are being more and more occupied with technology and the necessity to multitask. When faced with a situation involving trust, fewer resources are available to calculate probabilities concerning possible outcomes in a specific situation and, in consequence, a decision to express trust is less likely. Our results show that the observed decrease of trust may be yet another side effect of the social and technological development of societies.
